# Exploratory Patterns of Task Difficulty and Visual Feedback in Virtual Reality–Based Upper-Limb Trajectory Training

**DOI:** 10.2196/94745

**Published:** 2026-08-21

**Authors:** Yu Shi, Sophie Dewil, Zachary Marvin, Noam Y Harel, Raviraj Nataraj

**Affiliations:** 1 Department of Biomedical Engineering, Stevens Institute of Technology, 1 Castle Point Terrace, Hoboken, NJ, 07030, United States, 1 2012163555; 2 Movement Control Rehabilitation (MOCORE) Laboratory, Altorfer Complex, Stevens Institute of Technology, Hoboken, NJ, United States; 3 Spinal Cord Damage Research Center, James J. Peters Department of VA Medical Center, New York, NY, United States; 4 Department of Rehabilitation and Human Performance, Icahn School of Medicine at Mount Sinai, New York, NY, United States; 5 Department of Neurology, Icahn School of Medicine at Mount Sinai, New York, NY, United States

**Keywords:** virtual reality training, augmented sensory feedback, task difficulty, motor learning, upper extremity function

## Abstract

In this pilot evaluation of a virtual reality platform for upper-extremity motor rehabilitation, 9 neurotypical participants performed a force-modulated tracking task across 4 trajectory difficulty levels with and without augmented visual feedback; tracking accuracy improved in all conditions, with the descriptive visual-feedback advantage more pronounced at higher difficulty.

## Introduction

Virtual reality (VR)–based motor training provides a controlled and interactive environment from which to assign training task elements that shape movement practice and motor performance [1]. Such functionality with VR platforms is especially beneficial in designing protocols for motor rehabilitation [2]. This pilot study examined changes in force-based motor performance across two adjustable elements in VR-based training: augmented sensory feedback (ASF) and task difficulty. ASF provides additional real-time information about movement performance to support improved error correction [3]. Visual ASF is well-suited to immersive VR environments and can produce significant performance gains in VR task platforms [3]. The value of augmented feedback may increase with task demands, as greater path curvature and arc length raise spatial control requirements and reliance on external visual guidance [4]. As such, task difficulty is another essential training element, readily controllable in VR, and is central to ensuring skill acquisition in relation to user capability [5].

This pilot study presents a new version of a previously described VR training platform [6] that has participants exert muscular effort with the upper extremity to command a virtual avatar to trace target trajectories. A trajectory-based approach requires spatial accuracy while preserving user autonomy during continuous upper-limb VR training [7]. Further, this study newly explores target-tracking accuracy as a function of increasing training difficulty levels (ie, larger trajectory amplitudes) with and without augmented visual feedback. This pilot study was conducted with a small cohort of neurotypicals to demonstrate initial feasibility; however, the long-term application of this platform is VR-based motor rehabilitation of upper-extremity function after neurological injury [1].

## Methods

### Ethical Considerations

This study was approved by the institutional review board of Stevens Institute of Technology (protocol number 2021‐036). All participants provided written informed consent before participation.

### Participants and Experimental Design

**Figure 1.**
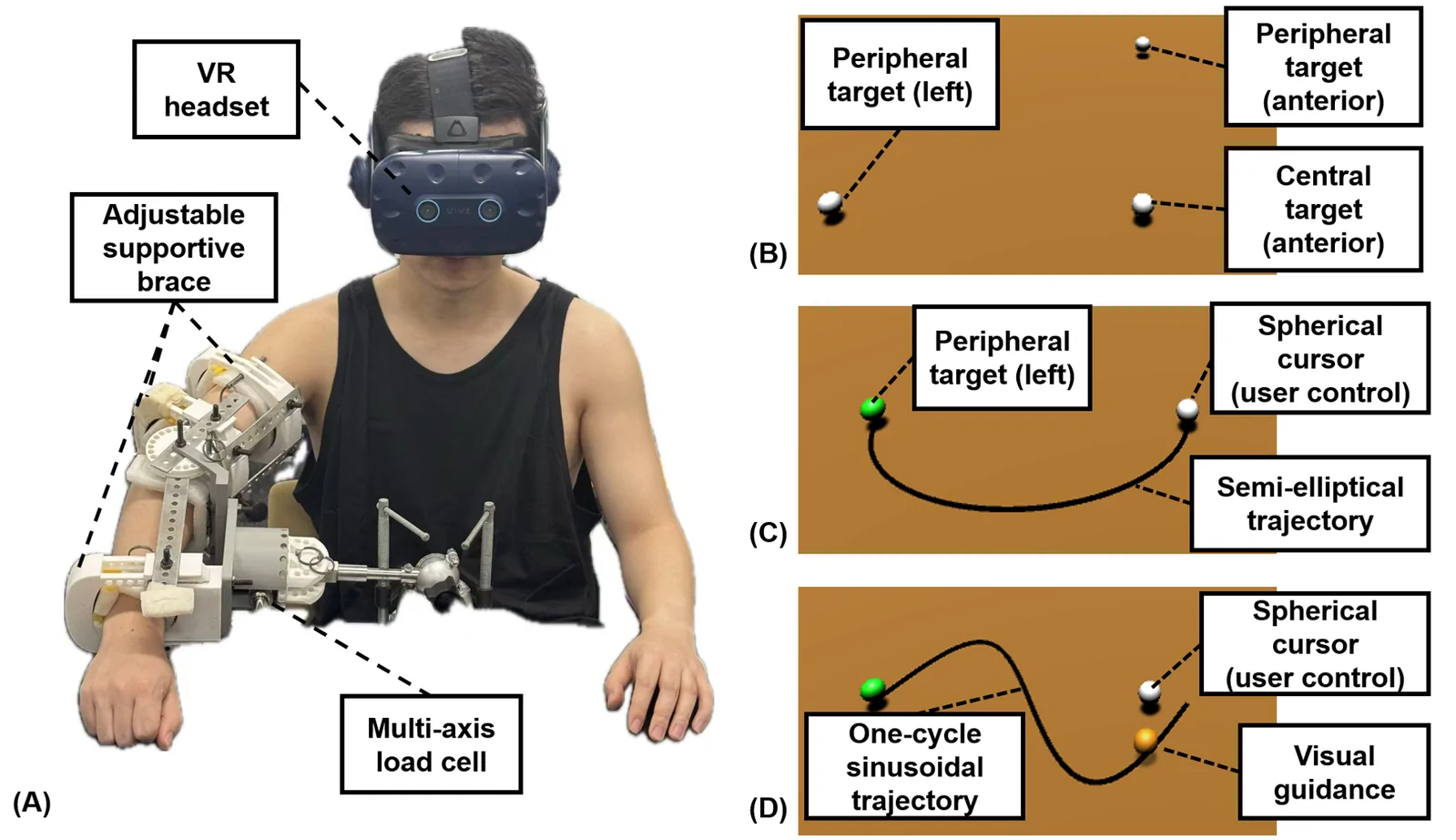
Experimental setup and VR task environment: (A) experimental general setup, (B) spatial arrangement of targets in VR space, (C) VR task environment in pre-/post-training session, (D) VR task environment in training session (with visual augmented sensory feedback and level 2 sinusoid). VR: virtual reality.

Nine neurotypical, right-side–dominant participants (5 males and 4 females; age range from 18 to 22 years) were enrolled in this study. The sample size reflected an exploratory repeated-measures pilot design rather than powered confirmatory testing. Participants were seated and placed their right arm in a position-adjustable brace used with a previously developed VR platform for training upper-limb function [6]. The virtual task environment was developed in Unity (Unity Software Inc) and displayed through an HTC Vive headset (HTC Corporation). A major adaptation of the prior platform was the addition of a tri-axial load cell (Fibos FA703) mounted to the brace to record 3D quasi-isometric forces. Only anterior-posterior (x) and medial-lateral (y) force components controlled the position of a spherical cursor in the x-y plane of the virtual environment (Figure 1A).

The task space contained one central target and two peripheral targets, which were contacted by the cursor after participants followed prescribed trajectories. Distance values were arbitrary Unity task-space units and did not correspond to real-world distances. Peripheral targets were 0.5 units in diameter and positioned 7 units from the central target (Figure 1B). In each trial, participants guided the cursor along the displayed trajectory to contact the active target; after a 1-second pause, the next target and trajectory appeared. Each peripheral target was activated twice in randomized order, followed by a return-to-center movement. Each condition included two pretraining, three training, and two posttraining trials. Pre/post trials used a semielliptical target trajectory (Figure 1C), whereas training trials used a one-cycle sinusoidal trajectory (Figure 1D), allowing assessment of generalization to an untrained but related movement path [8].

Participants completed 8 randomized training conditions (2 ASF conditions × 4 difficulty levels) in a single session, with 5-minute rests between conditions. Each condition took approximately 10 minutes, and no participant reported postsession fatigue. The ASF conditions were no ASF (control case, NF) and visual-only ASF (VF). Visual ASF was presented as a semitransparent orange sphere at the desired cursor location, defined as the projection of the current cursor position onto the prescribed trajectory (Figure 1D). Its position and opacity were continuously updated during movement; it appeared when tracking error exceeded 0.5 units, with opacity increasing linearly to fully opaque at 2.0 units. Difficulty levels L1-L4 used sinusoidal amplitudes of 1, 2, 3, and 4 Unity units, respectively. Motor performance (accuracy) was assessed according to tracking error, computed as the nonnormalized mean Euclidean distance between actual and desired cursor positions across sampled frames. Before calculating pretraining and posttraining mean tracking errors, contact-level tracking-error values were screened separately within each experimental condition using the rmoutliers function in MATLAB R2023b (MathWorks), with values more than 2 SDs from the corresponding condition mean removed. This screening was applied once, at the contact level; all participant-level values, VF – NF differences, and model inputs derive from these screened data. The effect of training on tracking accuracy was calculated as [(pretraining error − posttraining error) / pretraining error] × 100, representing the percentage change in performance from before to after training. As exploratory analysis, the difference in performance was evaluated between VF and NF conditions pooled across all difficulty levels. Further, an exploratory mixed effects model was fitted to examine if participant-level VF – NF differences were a function of difficulty level as a continuous fixed effect and participant modeled as a random intercept.

## Results

**Figure 2.**
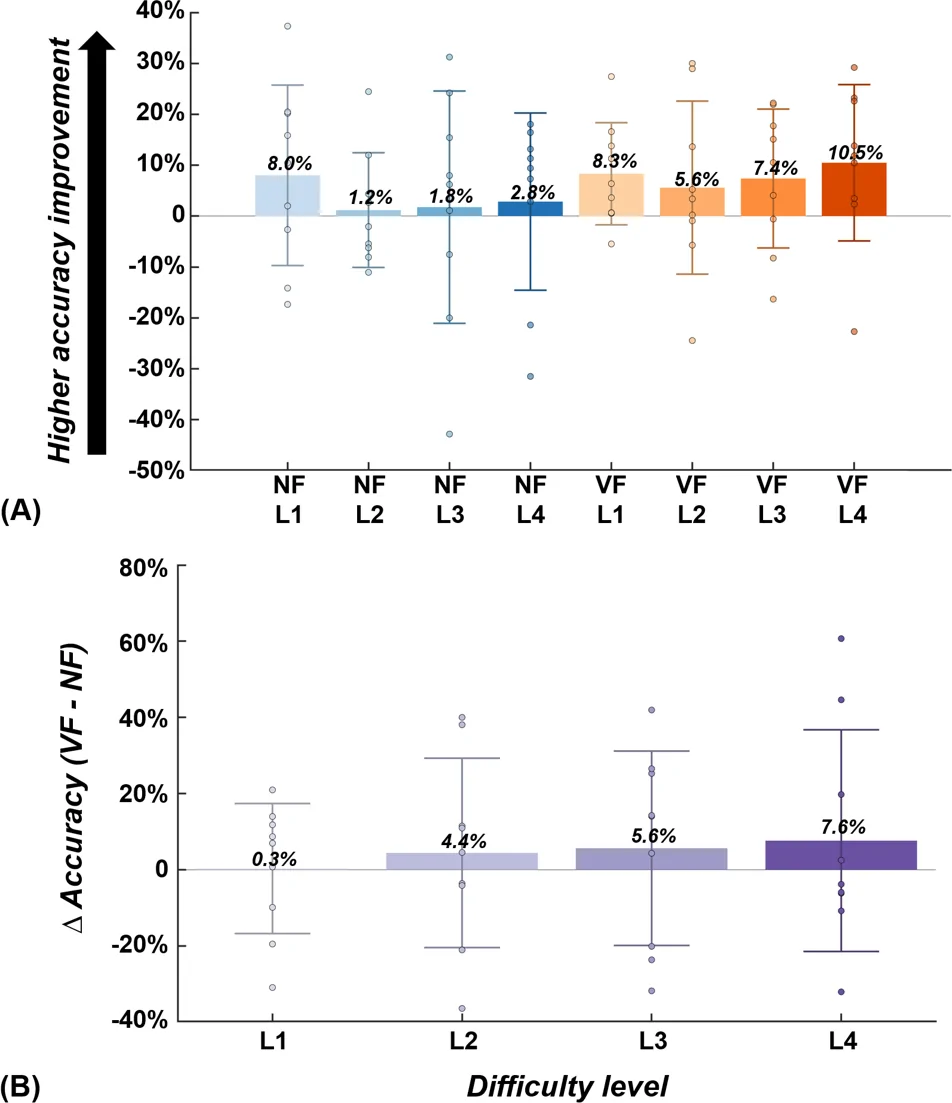
(A) Tracking accuracy improvement after training across ASF modes and difficulty levels. Bars show the percentage reduction in posttraining tracking error relative to pretraining error, with positive values indicating improved accuracy. Error bars indicate standard deviations across participants, and points represent individual participants. (B) Participant-level differences in tracking accuracy improvement between visual feedback and no feedback (VF – NF) across difficulty levels. Bars indicate group means, error bars indicate standard deviations across participants, and points represent individual participants. Positive values indicate greater improvement under VF than NF. ASF: augmented sensory feedback; L: difficulty level (1 to 4, increasing difficulty); NF: no augmented feedback; VF: visual augmented feedback.

Net percentage change in tracking accuracy was positive for all 8 conditions (Figure 2A). Under NF, the largest mean improvement occurred at the lowest difficulty level, L1, whereas improvements at higher difficulty levels were smaller. In contrast, VF produced positive improvements across all difficulty levels, ranging from approximately 5.6% to 10.5%. Across L1-L4, VF exceeded NF by approximately 4.5% on average. This descriptive difference became more pronounced when only L2-L4 were evaluated, with VF exceeding NF by approximately 5.9% (Figure 2B). An exploratory mixed effects model using participant-level VF – NF differences did not confirm a feedback-by-difficulty effect, with a positive but uncertain slope of 2.32% per difficulty level, a confidence interval spanning zero (–4.16 to 8.80), and an exploratory *P* value of .47. Thus, the pattern was interpreted as descriptive and hypothesis-generating. Processed participant-level mean tracking-error values for each condition are provided in Table S1 in Multimedia Appendix 1.

## Discussion

This pilot study examined how two essential VR training design elements (ASF and task difficulty) may interact to influence motor performance during trajectory tracking. Across conditions, tracking accuracy generally improved after training, with greater improvement when visual ASF was present, consistent with our prior VR studies [3,6]. The relatively highest increase with no ASF was observed at L1; however, reliance on lower-difficulty practice can limit progression and underrepresent the control demands required for learning more complex movements [9]. In this pilot study, the benefits of visual ASF were consistently amplified at higher difficulty levels (L2 to L4) and suggest that the benefit of ASF guidance during training increases with motor challenge. Further, our findings indicate the possibility that the difference between visual ASF to no ASF increases proportionately with difficulty level, suggesting visual ASF may either offset fatigue or facilitate greater engagement in the face of greater training challenge. Mechanistically, higher-difficulty trajectories may raise spatial control demands through greater curvature and path-following complexity, where real-time visual guidance becomes an especially useful external reference because the margin for spatial error narrows at higher difficulty. Together, these descriptive patterns offer preliminary rationale for future studies on how visual guidance cues and trajectory difficulty might be jointly tuned in trajectory-training task design [10].

Key study limitations include the small sample size, which was not powered for confirmatory inference, as well as the neurotypical participant sample, single-session design without retention testing, evaluation of only one sensory modality (visual ASF), lack of transfer to activities of daily living, and the absence of formal measures for fatigue or engagement. Thus, the results in this preliminary study are presented as descriptive and treated as hypothesis-generating. Future studies should use adequately powered designs and extend testing to individuals with neurological impairments, with longer-term work potentially exploring adaptive paradigms that adjust parameters such as visual ASF and task difficulty.

## Supplementary material

10.2196/94745 Multimedia Appendix 1 Participant-level mean tracking error values across training phases and conditions.

## Abbreviations

ASF: augmented sensory feedback
NF: no ASF
VF: visual-only ASF
VR: virtual reality

## References

[1] Soleimani M, Ghazisaeedi M, Heydari S (2024). The efficacy of virtual reality for upper limb rehabilitation in stroke patients: a systematic review and meta-analysis. BMC Med Inform Decis Mak.

[2] Dewil S, Kuptchik S, Liu M (2023). The cognitive basis for virtual reality rehabilitation of upper-extremity motor function after neurotraumas. J Multimodal User Interfaces.

[3] Sanford S, Collins B, Liu M, Dewil S, Nataraj R (2022). Investigating features in augmented visual feedback for virtual reality rehabilitation of upper-extremity function through isometric muscle control. Front Virtual Real.

[4] Sigrist R, Rauter G, Riener R, Wolf P (2013). Augmented visual, auditory, haptic, and multimodal feedback in motor learning: a review. Psychon Bull Rev.

[5] Guadagnoli MA, Lee TD (2004). Challenge point: a framework for conceptualizing the effects of various practice conditions in motor learning. J Mot Behav.

[6] Nataraj R, Liu M, Shi Y, Dewil S, Harel NY (2026). Training-induced modulation of motor task performance and psychophysiological domains through augmented sensory feedback in virtual reality. Front Virtual Real.

[7] Zhang L, Guo S, Sun Q (2020). Development and assist-as-needed control of an end-effector upper limb rehabilitation robot. Applied Sciences.

[8] Kawano T, Kouzaki M, Hagio S (2024). Generalization in *de novo* learning of virtual upper limb movements is influenced by motor exploration. Front Sports Act Living.

[9] Thomas A, Paul L, Rasenyalo S, Jones B, Hendricks S (2025). Challenge accepted: a systematic scoping review of the applications of the challenge point framework. J Mot Behav.

[10] Kleim JA, Jones TA (2008). Principles of experience-dependent neural plasticity: implications for rehabilitation after brain damage. J Speech Lang Hear Res.

