# Supplementary material for: Exploratory Patterns of Task Difficulty and Visual Feedback in Virtual Reality–Based Upper-Limb Trajectory Training
Source: JMIR XR Spat Comput. 2026 Aug 21;3:e94745. doi: 10.2196/94745 (PMC13496318; doi:10.2196/94745)
Supplement: Supplementary file 1 [file xr-v3-e94745-s001.docx]

**Table S1. Participant-level mean tracking error values across training phases and conditions.**

**Table S1A. Pre-training tracking error**

| Subject Number | NF-L1 | NF-L2 | NF-L3 | NF-L4 | VF-L1 | VF-L2 | VF-L3 | VF-L4 |
| --- | --- | --- | --- | --- | --- | --- | --- | --- |
| 1 | 0.5645 | 0.4161 | 0.3934 | 0.5052 | 0.4240 | 0.2973 | 0.3832 | 0.3096 |
| 2 | 0.3931 | 0.3517 | 0.3503 | 0.2695 | 0.3223 | 0.4046 | 0.6249 | 0.6240 |
| 3 | 0.5121 | 0.5053 | 0.4857 | 0.5258 | 0.5216 | 0.4407 | 0.5695 | 0.4437 |
| 4 | 0.4612 | 0.5419 | 0.6036 | 0.7264 | 0.4739 | 0.4588 | 0.5046 | 0.5508 |
| 5 | 0.6463 | 0.6028 | 0.5621 | 0.5155 | 0.4695 | 0.5023 | 0.4774 | 0.6273 |
| 6 | 0.5260 | 0.5324 | 0.6528 | 0.5624 | 0.5557 | 0.8561 | 0.4458 | 1.1085 |
| 7 | 0.4382 | 0.4226 | 0.4506 | 0.5188 | 0.5441 | 0.4468 | 0.5594 | 0.6755 |
| 8 | 0.3147 | 0.4127 | 0.3949 | 0.4003 | 0.3466 | 0.3755 | 0.3983 | 0.3911 |
| 9 | 0.5584 | 0.8983 | 0.7728 | 0.5963 | 0.8138 | 1.0485 | 0.9064 | 0.8417 |

**Table S1B. Training tracking error**

| Subject Number | NF-L1 | NF-L2 | NF-L3 | NF-L4 | VF-L1 | VF-L2 | VF-L3 | VF-L4 |
| --- | --- | --- | --- | --- | --- | --- | --- | --- |
| 1 | 0.3776 | 0.5153 | 0.6923 | 0.9744 | 0.3361 | 0.4136 | 0.5155 | 0.7843 |
| 2 | 0.2766 | 0.4485 | 0.7278 | 0.9167 | 0.3537 | 0.6326 | 0.9780 | 1.5438 |
| 3 | 0.3365 | 0.6063 | 0.8991 | 1.2217 | 0.4619 | 0.6304 | 0.9871 | 1.1260 |
| 4 | 0.3907 | 0.6366 | 1.0273 | 0.9635 | 0.4157 | 0.6348 | 0.9553 | 1.1537 |
| 5 | 0.5696 | 0.7785 | 0.9796 | 1.1787 | 0.4583 | 0.7267 | 0.9948 | 1.8642 |
| 6 | 0.3772 | 0.5920 | 0.8181 | 1.3954 | 0.3253 | 0.7735 | 0.6354 | 1.1151 |
| 7 | 0.3741 | 0.5931 | 0.8945 | 1.1250 | 0.4409 | 0.5423 | 0.8320 | 1.1091 |
| 8 | 0.2502 | 0.3601 | 0.4656 | 0.7074 | 0.2680 | 0.4033 | 0.4955 | 0.8083 |
| 9 | 0.5200 | 1.1539 | 1.1514 | 1.2716 | 0.6450 | 1.0161 | 1.4080 | 1.6516 |

**Table S1C. Post-training tracking error**

| Subject Number | NF-L1 | NF-L2 | NF-L3 | NF-L4 | VF-L1 | VF-L2 | VF-L3 | VF-L4 |
| --- | --- | --- | --- | --- | --- | --- | --- | --- |
| 1 | 0.3538 | 0.3662 | 0.4721 | 0.4387 | 0.3969 | 0.3699 | 0.2991 | 0.3023 |
| 2 | 0.3521 | 0.3709 | 0.3767 | 0.3272 | 0.3206 | 0.4084 | 0.5141 | 0.4792 |
| 3 | 0.4309 | 0.5157 | 0.4555 | 0.4763 | 0.4351 | 0.4658 | 0.5096 | 0.5443 |
| 4 | 0.5412 | 0.5757 | 0.8621 | 0.6732 | 0.4568 | 0.4348 | 0.5869 | 0.5315 |
| 5 | 0.5158 | 0.4554 | 0.4755 | 0.4225 | 0.4663 | 0.4853 | 0.5168 | 0.5529 |
| 6 | 0.4184 | 0.5754 | 0.4489 | 0.5464 | 0.4034 | 0.5993 | 0.4484 | 0.8580 |
| 7 | 0.4498 | 0.4112 | 0.4457 | 0.6821 | 0.4826 | 0.3859 | 0.4750 | 0.4783 |
| 8 | 0.3592 | 0.3947 | 0.2993 | 0.3346 | 0.3655 | 0.3747 | 0.3821 | 0.3501 |
| 9 | 0.5472 | 0.9976 | 0.7111 | 0.5289 | 0.7017 | 0.7450 | 0.7050 | 0.7255 |
